# The influence of employees' perception of over-qualification on career compromise: Mediated by role conflict and sense of relative deprivation

**DOI:** 10.3389/fpsyg.2022.1039800

**Published:** 2023-01-17

**Authors:** Xiaogang Zhou, Yunxi Zhang, Yanyan Lin, Liqing Li

**Affiliations:** ^1^School of Economics and Management, East China Jiaotong University, Nanchang, Jiangxi, China; ^2^School of Economics and Management, Jiangxi Science and Technology Normal University, Nanchang, Jiangxi, China

**Keywords:** career compromise, role conflict, relative deprivation, structural equation model, perception of over-qualification

## Abstract

In the external environment with the increasing level of education, there is a general phenomenon of excess qualification in the employment market. This research discusses employee career compromise from the perspective of employee over-qualification based on resource conservation theory and self-regulation theory. Combined with the survey data, a structural equation model (SEM) is constructed, and the mediation effect of relative deprivation and role conflict is analyzed according to the causal mediation model. The research find that employees' perception of over-qualification has three ways to affect employees' career compromise. First, employees' perception of over-qualification has a significant positive impact on their career compromise behavior through employees' emotions and self-cognition. Second, role conflict plays a partial intermediary role between the perception of over-qualification and career compromise by positively affecting career compromise behavior. Third, the sense of relative deprivation plays a partial intermediary role between the perception of over-qualification and career compromise by negatively affecting career compromise behavior. According to the research conclusions, the following suggestions are put forward. Enterprises need to establish a scientific employment mechanism to achieve talent-post matching and fundamentally reduce the phenomenon of over-qualifications. The company should pay attention to employee training, actively guide employees' career planning, instruct employees to correctly understand the sense of over-qualification and play a positive role in guiding employees' career planning.

## 1. Introduction

According to existing research reports, the proportion of Chinese employees who are considered overqualified is as high as 84% compared with the global average of about 44% (Ding X. L. et al., 2019), which is significantly higher than the average level, indicating that the phenomenon of over-qualification is becoming more common in China. How to minimize the negative impact of over-qualification on individuals and organizations has aroused widespread concern. Because the inconsistency between self-cognition and work environment will lead to low job satisfaction, high anxiety, conflict, high sense of deprivation, high burnout rate, and other negative emotions (Aranega et al., 2020; Zeng et al., 2020; Chen et al., 2021). These emotions are not only unfavorable to employees' proactive behaviors such as knowledge sharing, voice behavior and innovation (Zhao et al., 2015; Erdogan et al., 2020; Zhou et al., 2020; Zhao and Li, 2022), but also lead to employees' slacking behavior, withdrawal behavior, surface acting, and counterproductive work behavior (Liu et al., 2015; Andel et al., 2021; Cheng et al., 2021; Zhou and Wang, 2021). At the same time, the sense of overqualification forces the job seekers to fail to get the ideal job due to the differences between their own conditions and job opportunities, self-expectations, and the reality. In this case, it is often necessary to achieve the employment purpose through career compromise, and choose a job that is not very satisfactory (Gottfredson, 1981). It can be said that career compromise is a process that most people have to go through in the job search, and its consequences should not be underestimated. Creed and Blume (2013) found that career compromise has a negative predictive effect on individual career satisfaction, while it has a positive predictive effect on individual career dilemma and career compromise leads to job seekers' perception of their employability being reduced.

As a branch of the research field of career decision making, career compromise is the foundation and key of career decision making and an effective solution for individuals to solve the dilemma of career decision making (Super, 1953). It has been widely concerned by scholars since it was proposed (Gottfredson, 1981; Creed et al., 2020). Since the introduction of career compromise, researchers have mainly studied the antecedents and after-effects of career compromise from the perspectives of individual internal, external, goal setting, and social cognition (Zhang et al., 2022). However, there is a lack of sorting out the variables and mechanism based on the logical chain of “compromise inducement—compromise psychology—compromise behavior” (Zhang et al., 2020). This is not conducive to grasp the mechanism of career compromise caused by over-qualification psychologically and put forward targeted suggestions on the employment situation. Especially in the context of public health emergencies represented by the novel coronavirus pandemic, the employment situation has become more severe. The number of enterprises recruiting decreased significantly, resulting in limited job opportunities, enterprises choosing better quality employees from high-quality employees and the devaluation of diplomas, which made the sense of employee overqualification more frequent. To some extent, it affects the employment rate and employment quality, the effective allocation of human resources and the career growth of young talents (Liu et al., 2022). In order to solve this problem, it is very necessary to conduct more in-depth research on career compromise behavior from the perception of over-qualification. In addition, studying and solving the mechanism of career compromise caused by over-qualification has an important effect on a series of social problems such as the realization of personal career development and the alleviation of social employment pressure for the initial employment and re-employment (Weng et al., 2018).

Based on conservation of resource theory and self-regulation theory, this study deeply explores the internal mechanism of the impact of over-qualification on employees' career compromise with relative deprivation and role conflict as intermediary variables and it puts forward feasible countermeasures and suggestions in order to help enterprises optimize the concept of employment, enable employees to achieve ideal employment, and improve employees' happiness at work. The advantages of this study may be as follows. First, this paper takes the perception of over-qualification as an antecedent variable to explore its influence on career compromise, enriches the research on antecedent variables of career. At the same time, role conflict and relative deprivation are introduced as employees' psychological perceptions to construct a mediation effect model. Second, this study divides academic qualifications into those with junior college degree or below and those with bachelor degree or above, and distinguishes the gender of employees, so as to determine the different influences of overqualification, role conflict and relative deprivation on career compromise in different educational backgrounds and genders. This is an area that no previous research has explored Third, career compromise as a variable centered on the employee's career, is different from previous studies that explore the relationship between employees' perception of over-qualification and career compromise from the perspective of organizational interests. This study treats the perception of over-qualification from the perspective of individual employees, and it is also a supplement to the perspective and outcome variables of the research on the perception of over-qualification.

## 2. Theoretical analysis and research hypothesis

### 2.1. Perception of over-qualification and employee career compromise

With the improvement of the global education level, the phenomenon of employee overqualification is becoming more and more common in enterprises. Over-qualification describes an employment situation in which an employee's education, experience, ability, and other qualifications exceed the requirements of his or her job. The perception of over-qualification refers to the study of employees' attitudes and behaviors toward employment from the perspective of employees' perception (Zhu et al., 2018). As individuals increasingly focus on their own growth, companies increasingly focus on talent acquisition, and the labor market becomes increasingly oversupplied, the perception of over-qualification will only occur more frequently in organizations (Li and Li, 2021). Therefore, in the process that the over-qualification gradually becomes unavoidable, the job seekers are often forced to fail to obtain ideal jobs due to various differences between their own conditions and job opportunities, their own needs and the real environment. In this case, job seekers usually need to make career compromise behavior to achieve the purpose of employment. Career compromise refers to the compromise made in the realization stage of career ambition due to the difference between the ideal and the reality (Gottfredson, 1981).

When employees have excess qualifications, their skills, experience, and knowledge cannot be fully utilized. This will not only waste the resources employees have, but also prevent employees from making full use of the resources they have to create more resources for themselves. In this case, employees will think that their resources have been lost (Gilboa et al., 2008). Based on the Conservation of Resources Theory COR (Hobfoll, 1989), over-qualified employees have no opportunity to use their knowledge and skills in work, so they feel frustrated and unchallenged, and then show work withdrawal behavior. And when employees face the loss or threat of their own resources, they will develop tension and pressure, and further develop negative reactions such as emotional exhaustion and burnout. These negative reactions will lead to negative emotions in employees' work behaviors and attitudes (Maltarich et al., 2011; Zhao and Liu, 2020; Qiao and Yang, 2021). This negative emotion caused by the perception of over-qualification will cause employees to question their own abilities and values. And employees may feel that they're not performing as well as they should (Huang and Peng, 2017; Yuan et al., 2018). As a result, employees' self-esteem and core self-evaluation of the organization will be reduced, which will lead them to re-evaluate their own resources and recognize the gap between reality and ideal (Li et al., 2021). At the same time, Self-regulation Theory (SR) (Bandura et al., 1980) points out that individuals can achieve personal goals by adjusting self-cognition, emotion, and other behaviors and narrowing the gap between reality and ideal according to the gap between reality and ideal. Perception of over-qualification means that there is a gap between the reality and ideal state perceived by employees, and employees will take the initiative to adapt or change the environment when facing this gap (Zhang et al., 2020). At this point, employees will conduct a new judgment of their own cognition. In this process, employees will readjust their criteria for job-hunting and incorporate the options other than the original ideal position into their career selection space in order to achieve a better employment status. At the same time, the negative work attitude caused by over-qualification will also have a negative impact on employees' job-hunting expectations, which will reduce employees' requirements for career choice and further promote employees' career compromise behavior (Gati and Winer, 1987; Cheng et al., 2017).

Combined with the existing research, the perception of over-qualification is a subjective perception that widely exists in employees today (Van Dijk et al., 2020). Its influence on career compromise is multiple and interactive and it is difficult to explain the mechanism with a single theory. Accordingly, we propose the following hypothesis.

**Hypothesis 1:** The perception of over-qualification positively affects employees' career compromise behavior.

### 2.2. Perception of over-qualification and sense of relative deprivation

Many employees will continue to enrich their professional knowledge and skills during the period of studying or just starting to work, in order to get satisfactory returns in the future. However, when they perceive that the current position cannot provide them with a platform for full display, their qualifications exceed the requirements of the position, and they cannot get the expected returns, they will think that their abilities are given a lower value, which will lead to a sense of relative exploitation and injustice (Zhang et al., 2019; Zhao et al., 2019; Garcia-Mainar and Montuenga-Gomez, 2020). In particular, the degree of relative exploitation and unfairness will be greater when one's own situation is compared with that of other superior individuals inside and outside the company.

According to the theory of relative deprivation, a sense of relative deprivation occurs when an individual compares what he has with other individuals, or compares his current state with his past or potential deserved state. Individuals will feel a sense of deprivation when there is a difference between what they expect and deserve (Crosby, 1984). According to this view, the theory of relative deprivation is essentially based on a comparative perspective. First, when employees make use of accumulated experience and other ways to enhance their ability to work in all aspects. They will have expectations for their current jobs and opportunities based on their existing qualification level, and compare them with previous jobs or with the positions of employees with similar qualifications to judge whether their expectations are satisfied. After comparing their own development and opportunities, employees with a sense of overqualification perceive that there is a gap between the real situation and the inner expectation. The cognitive deviation caused by this gap will lead to their own perception of inferiority and a sense of unfairness, and then produce a sense of relative deprivation. Second, after comparing the current state with the state achieved by giving full play to their knowledge and experience, overqualified employees find that there is a gap between the two. And they find themselves at a disadvantage, so employees feel deprived. The more obvious this gap is, the stronger the sense of deprivation they feel. In addition, the sense of injustice brought by the perception of over-qualification negatively affects the construction of employees' identity and positive self-meaning (Smith et al., 2012), which makes them even more unable to rebuild themselves in social comparison and exacerbates the sense of relative deprivation. In conclusion, the reason for employees' perception of over-qualification is that there is a gap between the jobs they actually have and the jobs they hope to get. This gap will promote employees' sense of relative deprivation, and the higher the employees' perception of over-qualification is, the more obvious the relative deprivation will be. Accordingly, we propose the following hypothesis.

**Hypothesis 2:** The perception of over-qualification positively affects employees' sense of relative deprivation.

### 2.3. Perception of over-qualification and role conflict

The perception of over-qualification is manifested in the mismatch between the demand of the position and the ability of employees, that is, the working ability of employees is better than the working requirements of their positions (Debus et al., 2020). In addition, the perception of over-qualification also reflects the mismatch between the needs of employees and the opportunities provided by the organization, that is, the tasks or opportunities assigned by the organization cannot meet the needs of employees to give full play to their abilities and obtain higher work status (Yuan et al., 2020). When employees feel overqualified in the organization, it means that the organization provides jobs for employees that do not meet their expectations and requirements. Therefore, there is a state in which role expectation and role behavior are incompatible. At this time, there is a gap between what the employees hope to get from the post and what the post can actually provide for them, which leads to conflicts. This conflict may result in intra-role conflict among employees. When this gap is more obvious, the conflict will be more intense, and the role conflict perceived by employees will be more obvious. Role conflict refers to the conflicts that occur when individuals assume multiple obligations due to their various overlapping roles in society. It can be divided into inter-role conflict (conflicts between different roles played by individuals) and intra-role conflict (conflicts caused by inconsistent social expectations and requirements for the same role) (Gilboa et al., 2008; Robinson, 2013; Cui et al., 2014). According to the role theory, when an individual assumes a social role, he also needs to meet the expectations of others for the role. When the expectations of others for the role are inconsistent with the perception of the individual for the role, he will feel the role conflict.

Qu and Gao (2013) proposed that when personal values are matched with the organization's value system, both parties reach an agreement on understanding and analyzing issues, which can reduce employees' uncertainty perception, enable employees to more accurately grasp the organization's dominant thinking framework, clearly understand the organization's role expectations for individuals, and thus reduce role conflicts. When they don't match, role conflict will increase. In addition, according to self-determination theory, individuals have needs for autonomy, relationship and competence, and satisfying these needs is conducive to achieving positive organizational results. When these psychological needs are blocked, individuals will develop in a negative direction or produce functional dysfunction and then appear role conflict (Deci and Ryan, 2001). Finally, employees' behavior is mostly the result of their interactions with the organization (Chiaburu et al., 2014). The organization provides employees with resources to meet their needs and development. Based on the principle of reciprocity, employees will give back to the organization with a positive attitude and behavior (Hu and Song, 2012). When the organization does not meet the job needs of employees, employees will also return to negative attitudes and behaviors. Therefore, it can be inferred that when the work situation fails to meet the expectations or needs of employees, role conflict will increase. Accordingly, we propose the following hypothesis.

**Hypothesis 3:** The perception of over-qualification positively affects employees' role conflict.

### 2.4. The mediating role of role conflict

When assuming a professional role, an employee needs to meet the company's expectations for the role. When the leader's expectations for the role are inconsistent with the individual's own perception of the role, he will feel the role conflict (Rizzo et al., 1970; Netemeyer et al., 1990). Role conflict will make the work interaction between leaders and subordinates full of uncertainty (Schaubroeck et al., 1989), induce subordinates' psychological pressure, consume their emotional resources, and thus lead to subordinates' emotional exhaustion (Posig and Kickul, 2003). When employees are in the situation of mental and emotional resource depletion, they tend to emphasize or adopt avoidance and withdrawal coping strategies and behaviors (Mobley, 1982; Berry et al., 2012). Role theory points out that role conflict refers to the internal or emotional contradictions and conflicts that occur when individuals play social roles (Rizzo et al., 1970). And when faced with conflicts, people tend to rely on the initial role to make decisions in order to maintain the consistency of their attitudes (Robinson, 1996). In the context of severe employment situation and widespread overqualification, highly educated employees have to engage in positions with low ability requirements. Faced with the role conflict generated by this phenomenon, employees are more inclined to take the career compromise behavior of withdrawal and avoidance.

According to the resource loss spiral of resource conservation theory, the initial resource loss will lead to further resource loss, and the development of resource loss spiral will be more rapid, and the negative impact will be more intense. In order words, when employees feel role conflict, the environment provided by the organization will bring an obstructive pressure to employees. This obstacle is consistent with the consequences of the obstacle brought by over-qualification to employees in the resource conservation theory, which will make employees think that their own resources have been lost. Therefore, it can be similarly argued that when employees feel role conflict and think their resources have been lost, they will make certain career compromise according to the extent of resource loss. In conclusion, this study suggests that over-qualification positively affects career compromise through role conflict.

A series of longitudinal studies have also confirmed that role conflict plays an intermediary role between many psychology and behavior. For example, Schaufeli et al. (2009) study finds that role conflict completely mediated the relationship between workaholic and job demand, as well as the relationship between job burnout and happiness. Madera et al. (2013) finds that role conflict plays an intermediary role between perceived diversity and job satisfaction by exploring how managers' perceived diversity environment affects their work experience. Lu et al. (2015) points out that gender role conflict plays a mediating role between parenting style and male adolescents' mental sub-health. Zhang and Yang (2015) holds that role conflict plays a partially mediating role between person-organization matching and job alienation. Wang et al. (2015) finds that role conflict plays an intermediary role in the impact mechanism of assessment purpose on the performance of leading cadres. Shin et al. (2020) confirms that role conflict plays a partial and sequential intermediary role between managers' job shaping and turnover intention by examining how managers' job skills have a negative impact on their turnover intention. Accordingly, we propose the following hypothesis.

**Hypothesis 4:** Role conflict plays a mediating role in the relationship between employees' perception of over-qualification and career compromise behavior.

### 2.5. The mediating role of relative deprivation perception

When individuals judge their own current situation, they often look for something associated with themselves as the evaluation standard. The psychological mechanism of relative deprivation is that individuals evaluate their own status through horizontal comparison and vertical comparison, and generate corresponding emotional states on the basis of status cognition (Han et al., 2021). Relative deprivation has an important impact on individual behavior, mainly including deviant behavior (violence, theft, counterproductive work behavior, etc.) and avoidance behavior (smoking, gambling, social isolation, etc.) (Xiong and Ye, 2016). Employees with a high sense of relative deprivation will have negative evaluation of their own work environment (Zheng et al., 2019), and have a negative understanding of the position of individuals in the organization, accompanied by a strong negative emotional state. This negative emotion not only affects the behavior pattern of employees at work and makes them tend to adopt negative coping methods, but also brings a sense of inequality to employees when compared with others. This negative emotion and sense of inequality can also have a negative effect on employees' self-awareness (Geng, 2019), which will lead to employees' wrong judgments about their own employment prospects, resulting in employees' hesitation and doubt when looking for a job, thus missing ideal opportunities and turning to looking for easier opportunities (Xiong et al., 2021). Career compromise is the compromise of job seekers to their ideal career choice after measuring their own situation and reality, and then incorporate more accessible but non-ideal career into their career choice. According to the resource desperate principle in the resource conservation theory, when facing the desperate situation of resource depletion, the self-protection defense mechanism of individuals will be triggered, and they will show some aggressive and irrational behaviors. Such avoidant and irrational behavior manifests as career compromise in employees with high levels of relative deprivation. Therefore, over-qualification will positively affect career compromise through relative deprivation.

Many scholars have proved that the sense of relative deprivation acts as an intermediary between many psychology and behavior (Ding Q. et al., 2019). Zhou et al. (2022) finds that the sense of relative deprivation played a mediating role between negative life events and mobile phone dependence when she explored the relationship and internal mechanism between social support and relative deprivation in negative life events and mobile phone dependence. Ye et al. (2021) finds that relative deprivation plays a mediating role between bullying and aggressive behavior of college students. Accordingly, we propose the following hypothesis.

**Hypothesis 5:** Relative deprivation plays a mediating role in the relationship between employees' perception of over-qualification and career compromise behavior.

Our model assumptions are presented in Table 1.

**Table 1:** Model assumptions.

**Hypothesis**	**Research hypothesis**
H1	The perception of over-qualification positively affects employees' career compromise behavior
H2	The perception of over-qualification positively affects employees' sense of relative deprivation
H3	The perception of over-qualification positively affects employees' role conflict
H4	Role conflict plays a mediating role in the relationship between employees' perception of over-qualification and career compromise behavior
H5	Relative deprivation plays a mediating role in the relationship between employees' perception of over-qualification and career compromise behavior
H6	Relative deprivation positively affects employees' role conflict

**Figure 1:**
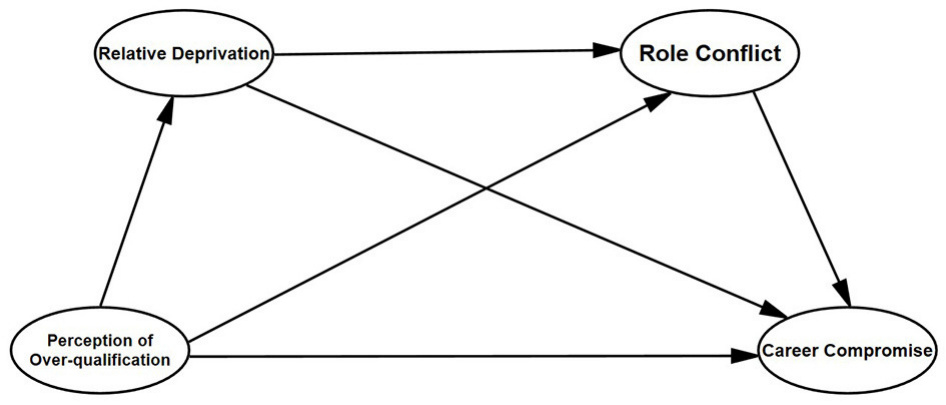
Research model.

Combined with research variables and research assumptions, the theoretical model of this study is specially constructed, as shown in Figure 1.

## 3. Materials and methods

### 3.1. Participants and procedure

The main research object of this study is the knowledge employees of enterprises. Data are collected by electronic questionnaire. With the help of MBA students from two universities in Jiangxi Province, we contacted six large-scale organizations (with more than 500 employees) located in Shanghai, Xi'an, Beijing, Hangzhou, and other places, mainly including public institutions, state-owned enterprises, and private enterprises. The employees are widely distributed, and the level difference is obvious. This study is based on stratified sampling of industry type and output size. Stratified sampling is adopted to collect samples from industries involved in new media, real estate, tourism, Internet, electric power, and transportation and issue questionnaires, which reduces the influence of the variability of each sampling layer and ensures that the samples are sufficiently representative. The survey was conducted from July 2021 to September 2021, and a total of 467 questionnaires were collected during that time period, excluding 37 invalid questionnaires (including those with obvious regularity in answers, too little or too much time to answer, obvious unreasonableness in the basic information section, and failure to complete the answers), a total of 430 valid questionnaires were obtained, and the effective rate was 92.1%.

The descriptive statistics results of the sample are as follows: 187 (43.5%) are male, 243 (56.5%) are female, making the proportion of men to women almost equal. In terms of educational background, 48 (11.2%) are in senior high school and below, 92 (21.4%) are junior college, 246 (57.2%) are undergraduate, and 44 (10.2%) are graduate and above. In terms of age, 122 (28.4%) are 23 years and below, 140 (32.6%) are 24–30 years old, 64 (14.9%) are 31–35 years old, 29 (6.7%) are 36–40 years old, 28 (6.5%) are 41–45 years old, 30 (7.0%) are 46–50 years old, and 17 (4.0%) are 51 years old and above. In terms of occupational levels, 26 (6.0%) are senior managers, 74 (17.2%) are middle managers, 127 (29.5%) are grass-roots managers, and 203 (47.2%) are other levels. In terms of length of service, 111 (25.8%) are within 1 year, 117 (27.2%) are 1–3 years, and 81 (18.8%) are 4–6 years, 39 (9.1%) are 7–10 years, and 82 (19.1%) are more than 10 years. In terms of monthly income, 103(24.0%) are 3,000 and below, 128 (29.8%) are 3,001–5,000, 141 (32.8%) are 5,001–10,000, 51 (11.9%) are 10,001–50,000, and 7 (1.6%) are 50,001 and above.

### 3.2. Measures

#### 3.2.1. Perception of over-qualification

According to the scale of perceived over-qualification developed by Maynard et al. (2006). The scale is divided into three topics: “perception of educational surplus,” “perception of skill excess,” and “over-experience perception.”

##### 3.2.1.1. Perception of educational surplus

In the design questionnaire of perception of educational surplus, the items for “The level of education required for my job is lower than my current academic qualifications,” “People with lower than my education can also do my current job well” and “My education level is higher than what my job requires.” Using Likert scale, options are “completely inconsistent (=1), somewhat inconsistent (=2), uncertain (=3), somewhat consistent (=4), completely consistent (=5).”

##### 3.2.1.2. Perception of skill excess

In the design questionnaire of perception of skill excess, the items for “Some of my job skills are not used in the current job position,” “My previous training is not of much use for this job” and “my ability is higher than what the job requires.” Using Likert scale, options are “completely inconsistent (=1), somewhat inconsistent (=2), uncertain (=3), somewhat consistent (=4), completely compliant (=5).”

##### 3.2.1.3. Over-experience perception

In the design questionnaire of over-experience perception, the items for “my previous work experience have little to do with my job,” “A lot of my knowledge is not needed in my current job” and “some people without my work experience can also do my current job well.” Using Likert scale, options are “completely inconsistent (=1), somewhat inconsistent (=2), uncertain (=3), somewhat consistent (=4), completely compliant (=5).”

#### 3.2.2. Relative deprivation

According to the scale of relative deprivation developed by Liang and Yue (2021). Using a cognitive and emotional two-dimensional structural model, the items of the questionnaire for “In my work, I actually get less than I should get,” “I was deprived of what I should have compared to people in the company who were similar to me,” “In my work, I got less than I expected,” “My work should be better rewarded than I do now,” “I can get more out of my job if something goes differently,” “I earn less than other people who are similar to me,” “I have a lower income compared to people who are similar to me,” “Compared to others, I am dissatisfied with the nature of the task I have taken on,” “Compared to others, I am dissatisfied with the attitude of the leadership toward me,” “Compared to others, I am frustrated with the situation I am facing now,” “The professional achievements of people in the company who are similar to me make me jealous” and “ I resent others for squeezing out what should have belonged to me at work.” Using Likert scale, options are “very inconsistent (=1), inconsistent (=2), somewhat inconsistent (=3), somewhat consistent (=4), consistent (=5), very consistent (=6).”

#### 3.2.3. Role conflict

According to the scale of role conflict developed by Rizzo et al. (1970), the items of the questionnaire for “At work I must do things in a way that is not what I think in my heart,” “At work I have to deal with some unnecessary things,” “At work I receive a task but there are not enough people to complete it,” “At work I receive a task but do not have enough resources and materials to perform it,” “At work I sometimes work with two or more teams that operate very differently,” “At work I have to break rules or policies in order to get things done,” “At work I get incompatible requests from two or more people” and “What I do at work is often accepted by individuals rather than multiple people.” Using Likert scale, options are “completely inconsistent (=1), relatively inconsistent (=2), somewhat inconsistent (=3), unclear (=4), somewhat consistent (=5), relatively consistent (=6), completely consistent (=7).”

#### 3.2.4. Career compromise

According to the scale developed by Weng et al. (2018), the scale of career compromise is divided into three dimensions: “development opportunity compromise,” “career matching compromise” and “social expectation compromise,” each dimension has four questions.

##### 3.2.4.1. Development opportunity compromise

In the design questionnaire, the items of the questionnaire for “Please recall carefully the degree of satisfaction with the opportunity to acquire new job skills in the future when considering whether to enter the current job position/occupation,” “Please recall carefully the degree of satisfaction with the opportunity to accumulate new work experience in the future when considering whether to enter the current job position/occupation,” “Please recall carefully the degree of satisfaction with the opportunity to learn new job knowledge in the future when considering whether to enter the current job position/occupation” and “Please recall carefully the degree of satisfaction with future promotion opportunities when considering whether to enter the current job/occupation.” Using Likert scale, options are “very dissatisfied (=1), not very satisfied (=2), uncertain (=3), relatively satisfied (=4), very satisfied (=5).”

##### 3.2.4.2. Career matching compromise

In the design questionnaire, the items of the questionnaire for “Please carefully recall that you are satisfied with the degree of use of the knowledge you have when considering whether to enter the current job position/occupation,” “Please recall carefully the degree of satisfaction with the degree of use of the job skills you have mastered when considering whether to enter the current job position/occupation,” “Please recall carefully the degree of conformity with your interests when considering whether to enter the current job position/occupation” and “Please carefully recall the degree of relevance to your career ideals and career goals when considering whether to enter the current job/occupation.” Using Likert scale, options are “very dissatisfied (=1), not very satisfied (=2), uncertain (=3), relatively satisfied (=4), very satisfied (=5).”

##### 3.2.4.3. Social expectations compromise

In the design questionnaire, the items of the questionnaire for “Please carefully recall the degree of satisfaction with your family's expectations when considering whether to enter the current job/occupation,” “Please carefully recall the degree of satisfaction with your friend's expectations when considering whether to enter the current job/occupation,” “Please recall carefully the level of satisfaction with the salary level offered when considering whether to enter the current job/occupation” and “Please recall carefully the degree of satisfaction with the social status corresponding to the work unit and the position when considering whether to enter the current job/occupation.” Using Likert scale, options are “very dissatisfied (=1), not very satisfied (=2), uncertain (=3), relatively satisfied (=4), very satisfied (=5).”

### 3.3. Reliability and validity test

Before the formal analysis of the sample data, the reliability and validity of the four scales of perception of over-qualification, relative deprivation, role conflict, and career compromise are tested. The Cronbach's α coefficient of each variable is calculated by SPSS 23.0 software. The specific results are show in the reliability analysis of the variables in Table 2.

**Table 2:** Reliability analysis of variables.

**Variable**	**Variable Cronbach's α**	**Item**
Perception of over-qualification	0.828	9
Relative deprivation	0.894	12
Role conflict	0.814	8
Career compromise	0.879	12
Population	0.957	41

It can be seen from the results in Table 3 that the Cronbach's α coefficient values of all variables are between 0.814 and 0.894, exceeding 0.8, indicating that the sample data reliability of the questionnaire is good. From the overall Cronbach's α coefficient value, it reaches 0.957, far more than 0.8, indicating that the scale has a certain good reliability, which provides a basis for further research.

**Table 3:** Validity result analysis.

**Variable**	**KMO-value**	**Approximate chi-square**	**df**	**Significance**
Perception of over-qualification	0.874	1024.623	36	0.000
Relative deprivation	0.918	2153.509	66	0.000
Role conflict	0.856	897.091	28	0.000
Career compromise	0.918	2153.509	66	0.000
Population	0.963	8140.868	82	0.000

Then the KMO value of each variable is calculated. The results are presented in Table 3 validity result analysis.

The data in Table 3 show that the corresponding probability *p*-value of the overall scale represented by each factor in the formal questionnaire is 0.000, which is less than the significance level a, and there is a significant difference. At the same time, the KMO values of all scales are higher than 0.8, and the validity of the questionnaire is good.

### 3.4. Common variance analysis

The collected data were tested using the Harman single factor method for the common variance in the study. The results showed that there were 6 factors with feature roots >1, and the first principal component obtained without rotation accounted for 37.56% (53.45% in total), not more than 40%. Incorporate the common variance factor as a latent variable into the structural equation modeling. The results show that the model fitting index is better after incorporating the common variance factor (χ^2^/*df* = 2.036, IFI = 0.929, CFI = 0.928, RMSEA = 0.042), but compared with the theoretical model, The key indicator changes are >0.03 (Δχ^2^/*df* = 0.022, ΔIFI = 0.008, ΔCFI = 0.007, ΔRMSEA = 0.008). In view of this, there are no significant common variance in this study.

### 3.5. Ethics statement

This study was conducted in accordance with the Declaration of Helsinki, and the protocol was approved by the Ethics Committee (HREC) of the School of Economics and Management in East China Jiaotong University. The participants provided their written informed consent to participate in this study.

## 4. Results

### 4.1. Model fit degree analysis

It can be seen from the fitting index value of the structural equation model in Table 4 that the structural equation model fit index, the model fit index meet the evaluation criteria, and *X*^2^/*df* and RMSEA fit ideal. From the relative fitting index, the results of NFI, RFI, IFI, TLI, and CFI fit well. NFI and RFI reached 0.9, and the model fitting effect is good. The simplified fitting indexes can meet the evaluation criteria, and the results of PNFI, PCFI, and PGFI fit well.

**Table 4:** Model fitting index values.

**Statistical tests**	**Indicators**	**Evaluation criterion**	**Model results**	**Fitting**
Absolute fitness index	CMIN/DF	≤ 5.0	2.058	Ideal
	RMSEA	≤ 0.08	0.050	Ideal
Value-added fitness index	NFI	≥0.90	0.925	Ideal
	RFI	≥0.90	0.935	Ideal
	IFI	≥0.90	0.921	Ideal
	TLI	≥0.90	0.914	Ideal
	CFI	≥0.90	0.921	Ideal
Minimalist fitting index	PGFI	≥0.50	0.738	Ideal
	PNFI	≥0.50	0.756	Ideal
	PCFI	≥0.50	0.830	Ideal

Overall, the structural equation model has good fitting.

### 4.2. Testing of hypotheses

Through the operation of structural equation model (SEM), the path coefficient level is analyzed to verify its effectiveness.

As shown by the model parameter estimation in Table 5, the perception of over-qualification has a positive and significant impact on employees' career compromise (β = 0.669, *p* < 0.05), and H1 is supported. The perception of over-qualification has a positive and significant effect on relative deprivation (β = 0.871, *p* < 0.05), and H2 is supported. The perception of over-qualification has a positive and significant impact on role conflict (β = 1.059, *p* < 0.05), H3 is supported. Role conflict has a positive and significant impact on employee career compromise (β = 0.214, *p* < 0.05), H4 is supported. The sense of relative deprivation has a positive and significant impact on employee career compromise (β = 0.213, *p* < 0.05), and H5 is supported. Relative deprivation has no significant effect on role conflict (β = 0.017, *p* > 0.05), H6 is not supported.

**Table 5:** Model parameter estimation.

**Variable**	**Standardized path coefficients**	**S.E**.	**C.R**.	***p*-value**	**Result**
Career compromise ←-- perception of over-qualification	0.701	0.195	2.815	0.005	Supported
Relative deprivation ←-- perception of over-qualification	0.937	0.120	9.459	^***^	Supported
Role conflict ←-- perception of over-qualification	0.877	0.277	4.467	^***^	Supported
Career compromise ←-- role conflict	0.635	0.140	2.521	0.012	Supported
Career compromise ←-- relative deprivation	0.425	0.136	2.025	0.043	Supported
Role conflict ←-- relative deprivation	0.150	0.203	−0.862	0.389	Unsupported

^***^p < 0.001.

**Figure 2:**
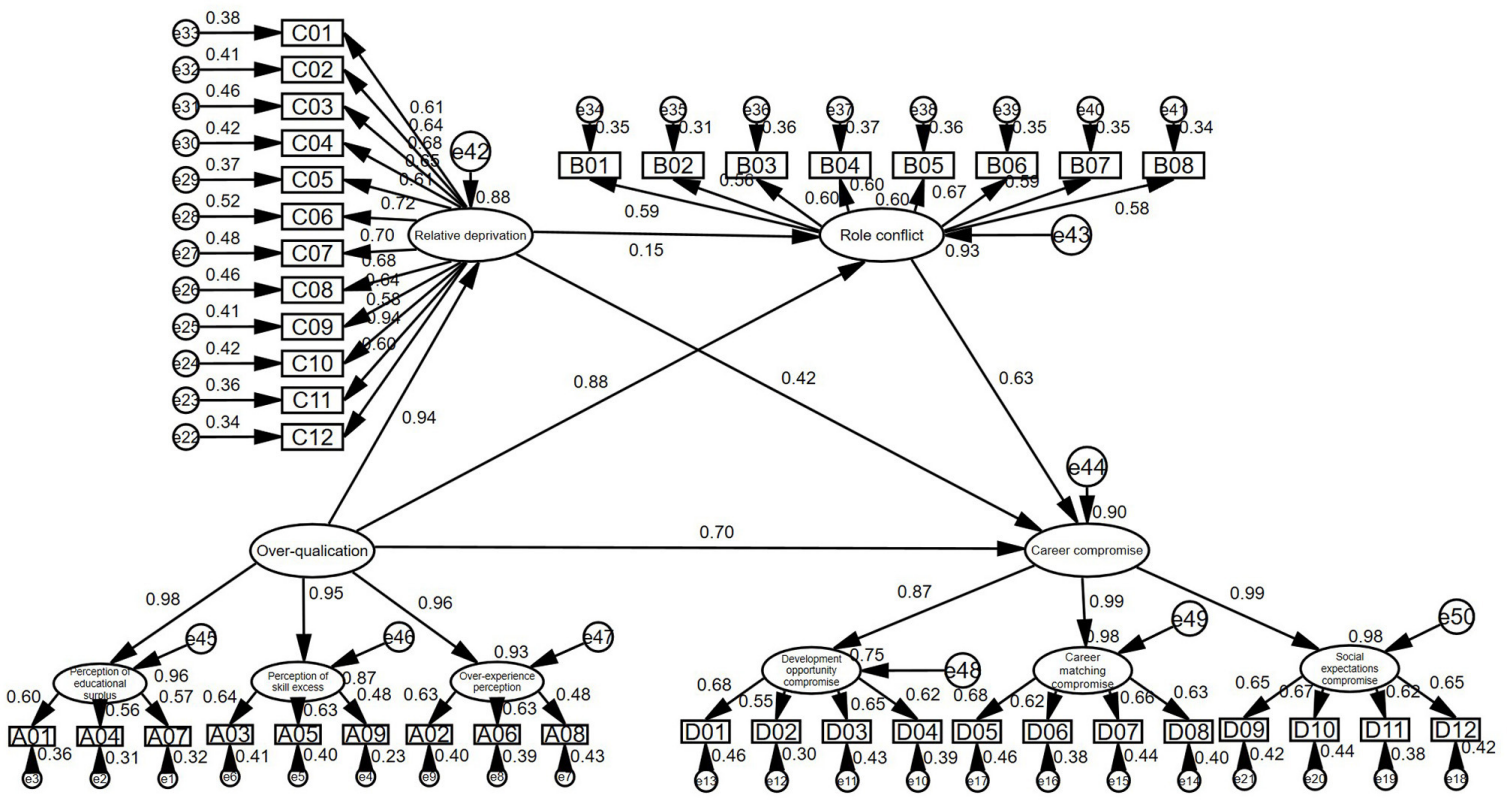
Statistical diagram of standardized path coefficient of structural equation model.

Figure 2 Standardized Path Coefficient Statistical Chart is the modified model diagram, and each path coefficient of the model is shown in the figure.

The hypotheses are tested by using structural equation modeling. According to the revised model diagram, the following views are also confirmed.

First, perception of over-qualification has a direct positive impact on employee career compromises. The path correlation coefficient between the two is 0.70, indicating that for every unit that the employee over-qualification perception, the employee career compromise will increase by 0.70 units. The more significant the perception of over-qualification, the higher the occupational compromise behavior generated by the employee. According to the theory of resource preservation and self-regulation, employees feel that excess qualifications will produce negative work attitudes and behaviors. This can cause them to doubt themselves, re-evaluate their resources, lower their job search standards and make compromises to the original ideal career choices, and include non-ideal occupations in the scope of career choice. Hypothesis 1 is tested.

Second, perception of over-qualification has a direct positive impact on the sense of employee relative deprivation. The path correlation coefficient between the two is 0.94, indicating that for every unit that the perception of employee over-qualification, the relative sense of deprivation will increase by 0.94 units. The more significant the perception of over-qualification, the higher the sense of relative deprivation. According to the theory of relative deprivation, when employees are compared with other employees with similar qualifications or with their previous jobs, they perceive that they are overqualified, and this sense of injustice will promote the sense of relative deprivation of employees. And the higher the perception of over-qualifications of employees, the more obvious their relative deprivation. Hypothesis 2 is tested.

Third, perceived of over-qualification has a direct positive impact on employee role conflict. The path correlation coefficient between the two is 0.88, indicating that for every unit that the employee over-qualification perception, the sense of role conflict will increase by 0.88 units. The more significant the perception of over-qualification, the higher the sense of role conflict. According to the theory of people-post matching, when employees feel over-qualifications in the organization, it means that the jobs provided by the organization to employees do not meet the expectations and requirements of employees, that is, there is a gap between what employees hope to get from the position and what the position can actually provide for employees, which in turn leads to conflicts between employees (Pandey and Kumar, 1997). Hypothesis 3 is tested.

Fourth, role conflict has a direct positive impact on employee career compromise. The path correlation coefficient between the two is 0.63, indicating that for every unit that the sense of employee role conflict is promoted, the behavior of career compromise will increase by 0.88 units. The more significant the sense of employee roles conflict, the higher the sense of career compromises. According to the theory of resource preservation, when employees feel conflicting roles, the environment provided by the organization will bring a kind of obstructive pressure to employees, they will think that their resources and abilities are exhausted in this environment, and they will make certain career compromises according to the degree of resource loss. Hypothesis 4 is tested.

Fifth, relative deprivation has a direct positive impact on employee career compromise. The path correlation coefficient between the two is 0.42, indicating that for every unit that the sense of employee relative deprivation, the employee career compromise will increase by 0.42 units. The more significant the sense of employee relative deprivation, the more the professional compromise it produces. When employees have a stronger sense of relative deprivation after comparing with superior individuals, their sense of inequality and negative perception will be enhanced. This sense of inequality will affect employees' judgment and emotions, causing them to miss the present opportunity, and consequently, employees will have more career compromise behaviors in their subsequent employment. Hypothesis 5 is tested.

Sixth, relative deprivation has no significant effect on employees' role conflict. The root cause of role conflict is that individuals assume two or more different roles at the same time, and the roles are incompatible (Zhao et al., 2021). However, by comparing the employees of the same level or the same type of work, the over-qualified employees find that the work does not meet their expectations, resulting in a feeling that they are relatively deprived of the position and salary they deserve, which leads to anger and resentment and counterproductive behavior, without triggering the generation mechanism of role conflict (Zhao and Peng, 2021). Hypothesis 6 is rejected.

Seventh, relative deprivation and role conflict play a mediating effect. In order to further verify the mediating effects of relative deprivation and role conflict, this study uses Amos software and deviation corrected bootstrap confidence interval method, and the confidence level is set to 95%.

According to the bootstrapping mediation effects testing in Table 6, the following conclusions can be drawn.

**Table 6:** Bootstrapping mediation effects testing.

**Summary of the hypothesized path**	**Coefficient**	**Bias-corrected 95% CI**		**Statistical significance**
		**LL**	**UL**	
Total effect	1.027^***^	0.568	5.173	Significant
Perception of over-qualification → Career compromise				
Direct effects	0.519^***^	0.119	1.716	Significant
Perception of over-qualification → Career compromise				
Indirect effects	0.553^***^	1.120	2.472	Significant
Perception of over-qualification → Relative deprivation → Career compromise				
Perception of over-qualification → Role conflict → Career compromise	1.061^***^	0.220	5.290	Significant

^***^p < 0.001. CI, confidence interval; LL, lower limit; UL, upper limit.

First, the bootstrap deviation correction confidence interval of the indirect effect of relative deprivation on the perception of over-qualification and the employee career compromise under 95% confidence is (−2.472, −0.120), zero is not within this range, and the *p* < 0.05. It shows that relative deprivation has a significant mediating effect between the perception of over-qualification and the employee career compromise. At the same time, the bootstrap deviation correction confidence interval of the direct effect of relative deprivation on the perception of over-qualification and the employee career compromise is (0.508, 0.611), zero is not within this interval, and the *p* < 0.05, indicating that relative deprivation plays a partial mediating effect between on the perception of over-qualification and the employee career compromise.

Second, the bootstrap deviation correction confidence interval of the indirect effect of role conflict on the perception of over-qualification and the employee career compromise under 95% confidence is (0.220, 5.290), zero is not within this range, and the *p* < 0.05. It shows that role conflict has a significant mediating effect between the perception of over-qualification and the employee career compromise. At the same time, the bootstrap deviation correction confidence interval of the direct effect of role conflict on the perception of over-qualification and the employee career compromise is (0.119, 1.716), zero is not within this interval, and the *p* < 0.05, indicating that role conflict plays a partial mediating effect between on the perception of over-qualification and the employee career compromise.

To sum up, according to the bootstrap test results, both Perception of over-qualification → relative deprivation → career compromise path and Perception of over-qualification → role conflict → career compromise path pass the significance test at the level of 0.01. It can be seen that the two intermediary paths are significant.

### 4.3. Comparison of differences between multi-group models

The fitting of the above model was based on the assumption of homogeneity for all groups. The internal structures of all the groups were heterogeneous. Therefore, it was necessary to compare the differences between the types of educational groups and groups of different gender to reveal the differences between different groups more intuitively and accurately. According to the education type and gender type, the sample is divided into college degree or below, bachelor degree or above, male and female. The classification of education types by the field of junior college can more intuitively distinguish the group with higher education and the group with lower education, making the research results more scientific and reasonable.

Table 7 reflects the degree of fit between different groups of models and survey data. The empirical results show that the fitting data of the three models of women with college degree or below and bachelor degree or above meet the evaluation criteria, indicating that the fitting index of the three models is relatively idea. However, in the male fitting model, the GFI and AGFI values are slightly lower than the evaluation criteria, indicating that the model is well-fitted. Therefore, the fitting levels of the four different groups of models constructed in this study are acceptable.

**Table 7:** Comparison of parameters between different groups.

**Variable**	**Different population**	**GFI >0.90**	**AGFI >0.90**	**RMSEA <0.08**	**CFI >0.90**	**Result**
Different education groups	Junior college and below	0.919	0.913	0.043	0.940	Ideal
	Bachelor degree or above	0.923	0.910	0.032	0.946	Ideal
Different genders	Male	0.892	0.878	0.076	0.912	Good
	Female	0.933	0.910	0.046	0.968	Ideal

“Ideal” means that the fitting index is within the reference value range; “good” means that the fitting index is not within the reference value range but is slightly lower or slightly higher.

From different education groups and gender groups, we compared the effects and path coefficients of overqualification, role conflict, relative deprivation and role conflict, as shown in Table 8. The coefficients in Table 8 are standardized.

**Table 8:** Comparison of path standardization coefficient between different groups.

**Path**	**Different education groups**		**Different genders**	
	**Junior college and below**	**Bachelor degree or above**	**Male**	**Female**
Career compromise ←-- perception of over-qualification	0.201^***^	0.321^***^	0.227^***^	0.389^***^
Relative deprivation ←-- perception of over-qualification	0.115^***^	0.414^***^	0.305^***^	0.394^***^
Role conflict ←-- perception of over-qualification	0.246^***^	0.251^***^	0.197^***^	0.431^***^
Career compromise ←-- role conflict	0.192^***^	0.221^***^	0.064^**^	0.374^***^
Career compromise ←-- relative deprivation	0.163^**^	0.177^**^	0.155^***^	0.225^***^
Role conflict ←-- relative deprivation	0.022	0.004	0.002	0.064^*^

^*^p < 0.05, ^**^p < 0.01, ^***^p < 0.001.

First, the influence coefficient of the perception of over-qualifications in the group with a bachelor's degree or above on career compromise is greater than that in the group with a college degree or below, and the influence coefficient of the perception of over-qualifications in the group with a bachelor's degree or above on role conflict and relative deprivation is greater than that in the group with a college degree or below. A possible reason is that people with a bachelor's degree or above have higher education, so the sense of overqualification will be higher than those with lower education, and people with higher education are better at judging the situation and self-regulation. Therefore, in the context of over-qualification, people with higher education are more likely to conduct self-cognition and adjustment, and then make career compromise behavior. However, in the face of the sense of relative deprivation and role conflict brought by over-qualification, the group with low education is often unable to carry out accurate self-evaluation and is more likely to be impulsive when making decisions. Second, the influence coefficient of women's perception of over-qualification on career compromise behavior is greater than that of men, and the influence coefficient of women's perception of over- qualification on role conflict and relative deprivation is greater than that of men. One possible reason is that some work arrangements (such as short-term reassignment) may occupy the time of female employees' family role and affect their work-family balance, which leads them to choose to sacrifice their career development and make professional compromise behavior to maintain their family (Shortland, 2015). Packard and Babineau (2009) found that individuals are more likely to make career compromises when faced with financial distress, limited time, heavy family responsibilities and weak vocational skills. Women have more responsibilities at home than men, and men's skill level in the workplace is often considered to be higher than that of women. Therefore, women are more likely to make career compromise behaviors in the face of a series of problems brought by over-qualification.

## 5. Discussion

This paper first analyzes the descriptive statistical characteristics of the sample, establishes a structural equation model to explore the impact of employee over-qualification on employee career compromise, and uses the bootstrap method of deviation correction to test the mediating effect between relative deprivation and role conflict on employee over-qualification perception and employee career compromise behavior. Through the research, the following basic conclusions are drawn: First, employees' perception of over-qualification has a significant positive correlation with their career compromise behavior. Based on conservation of resource theory and self-regulation theory, conservation of resource theory refers to the tendency of individuals to strive to acquire, cultivate, maintain, and protect important resources (Luksyte and Spitzmueller, 2016). When individuals face the loss or threat of their own resources, they will appear emotional exhaustion, burnout and other negative reactions, and appear negative behaviors such as aggression and irrationality to defend themselves (Hobfoll et al., 2018). At the same time, the self-regulation theory points out that individuals will narrow the gap between reality and ideal by adjusting their self-cognition, emotions, and other behaviors according to the gap between reality and ideal to achieve personal goals (Jaramillo et al., 2017). The perception of over-qualification will make employees have negative emotions, thus questioning their own ability and value, causing employees to re-evaluate their own resources, read just their job search standards, and then urge employees to make professional compromises. Second, employees' perception of over-qualification has a significant positive impact on relative deprivation and role conflict. By comparing with other individuals, or comparing their current status with their past or potential deserved status, employees will feel frustrated, and unfair when they feel that they are less than deserved. This sense of injustice brought by employees' perception of over qualifications leads to the generation of sense of relative deprivation. At the same time, when employees feel excess qualifications in the organization, it means that the working situation cannot meet the expectations or needs of employees, so there is a state of disharmony between role expectations and role behaviors. This state leads to role conflict among employees. Thirdly, relative deprivation and role conflict have a significant positive effect on employees' career compromise behavior, and they play a mediating effect in the mechanism of action. According to the conservation of resource theory, when employees feel role conflict, the environment provided by the organization will bring obstructive pressure to employees. They will think that their own resources and abilities are exhausted in such an environment, and they will make certain career compromises according to the degree of resource loss. When employees have a sense of inequality and relative deprivation, this sense of inequality will affect their judgment and emotions, causing them to miss the immediate opportunity, and thus they will have more career compromise behaviors in the future employment. These conclusions are generally consistent with previous research (Chen et al., 2020; Liu and Tang, 2022). In addition, there are significant differences in career compromise behavior among different attribute groups. The influence coefficient of female group's overqualification on career compromise, role conflict and relative deprivation is greater than that of male group. The influence of over-qualification perception on career compromise, role conflict and relative deprivation in the group with higher education is greater than that in the group with lower education.

## 6. Theoretical implications

The study makes the following theoretical contributions.

First, from the perspective of resource conservation theory, self-regulation theory, and relative deprivation theory, this study explains the influence mechanism of the perception of over- qualification, relative deprivation perception, and role conflict on career compromise behavior. In recent years, many scholars have begun to pay attention to the influence of over-qualification and relative deprivation on career compromise, but most of them are limited to a single study of over-qualification and career compromise or relative deprivation and career compromise. Lack of research on the integration of the three, its internal mechanism is not clear. This study not only reveals that the perception of over-qualification has a promoting effect on career compromise behavior, but also the mediating effect of relative deprivation and role conflict between the perception of over-qualification and career compromise behavior.

Second, while domestic and foreign scholars have conducted relevant studies on the antecedents and aftereffects of the perception of over-qualifications and career compromise (Creed and Gagliardi, 2015; Zhang et al., 2016), this research found that relative deprivation plays an important mediating role in the influence of perceived excess qualifications on career compromise behavior. This study not only included relative deprivation into the research framework of the influence of perceived excess qualifications on career compromise behavior, but also introduced role conflict variables to jointly explore the internal mechanism of the influence of perceived excess qualifications on career compromise behavior. The perception of over-qualification forces the job seekers to fail to get the ideal job due to the differences between their own conditions and job opportunities, self-expectations, and the reality. In this case, career compromise is often needed to achieve the purpose of employment, which means that they settle for the second best and choose a job that is not very satisfactory (Gottfredson, 1981). To sum up the current study, it expands the research context of the factors influencing career compromise and enriches and deepens research results related to career compromise.

Third, this study divides the group into male, female, highly educated, and poorly educated, in order to identify the different influences of the perception of over-qualification on the sense of relative deprivation, role conflict, and career compromise behavior of different types of employees. Specifically, the influence coefficient of the perception of over-qualification on the career compromise behavior of the highly educated group is higher than that of the low-educated group, and the perception of over-qualification on the career compromise behavior of women is higher than that of men. Highly educated people are better at measuring the situation and adjusting themselves. Therefore, in the context of excess qualifications, people with higher education are more likely to conduct self-cognition and adjustment, and then make career compromise behavior. However, in the face of the sense of relative deprivation and role conflict brought by excess qualifications, the group with low education is often unable to carry out accurate self-evaluation and is more likely to be impulsive when making decisions. Women have more responsibilities at home than men, and men's skill level in the workplace is often considered to be higher than women's. Therefore, women are more likely to make professional compromise behaviors when facing a series of problems brought by excess qualifications.

## 7. Implication for research and practice

How to reduce the negative impact of the perception of excess qualifications is the focus of increasing attention from all walks of life. According to the conclusion of this paper, the following suggestions are put forward.

First, we should achieve the matching of people and posts, and fundamentally reduce the phenomenon of over-qualifications. When recruiting talents, enterprises should not only pay attention to education background or school, but should seek suitable talents according to the needs of the position pragmatically. Enterprises also should improve the competency model of different positions to achieve the best man-post matching, so that employees' abilities can be fully displayed in the corresponding positions. And fundamentally reduce the phenomenon of overqualified and underemployed, so as to reduce the perception of overqualified within the company and weaken its possible negative impact on employees.

Second, formulate a hierarchical training system and do a good job in skill training. Enterprises should pay attention to quality development, so that employees have the opportunity to contact and master other skills. At the same time, it is necessary to strengthen the active guidance of employees' career planning, meet their career needs, and help employees realize their career aspirations in the organization while improving organizational performance and core competitiveness.

Third, employees should carry on the scientific and reasonable self-cognition adjustment. In the process of career planning, individual cognition plays an important role. The perception of over-qualification will have a certain negative impact on self-cognition, induce them to have a sense of job insecurity, and then show a higher career compromise. Employees should calmly and objectively judge, evaluate and summarize their own resources, treat their career planning scientifically and dialectically, revise their career goals, and reduce the destructive effect of perception of over-qualification.

## 8. Limitations and future research

This study also has the following limitations.

First, this study did not do the analysis of multiple time points. Some interview data show that over-qualified employees have experienced emotional fluctuations and behavioral changes, but this paper focuses on analyzing the current state and behavior of employees. In the future, longitudinal tracking cases or key events can be used to explore the dynamic changes in the work behavior of over-qualified employees.

Second, based on the perspective of resource preservation and self-regulation, this study explores the influencing mechanism of over-qualification and career compromise emotionally and psychologically. Future research can explore its internal mechanism from other perspectives, such as the perspective of paradox.

Third, the main variables involved in this study are all employees' individual perceptions, which cannot reflect the real situation of the organization. In future studies, variables at the organization or team level can be introduced to explore the relationship between employees' perception of over-qualification and career compromise.

## 9. Conclusion

The present study found that the perception of over-qualification can not only directly lead to professional compromise behavior, but also lead to the result of career compromise behavior through different psychological perception mechanisms—relative deprivation and role conflict. We found that the female group and the highly educated group have more sense of deprivation and conflict in the face of excess qualifications, and are also more likely to make career compromise behavior. In conclusion, this study is helpful to clarify the underlying psychological process of the performance of over-qualification as employees' career compromise behavior, and the model is fitted for different gender and educational background groups to draw corresponding conclusions. As such, our findings highlight meaningful directions for future conceptual and empirical work in this area, and provide important insight into how organizations can best grasp the psychology of different groups of employees and alleviate the associated negative behaviors caused by excess qualifications.

## Data availability statement

The datasets presented in this article are not readily available because of privacy protection regarding the data of the participants. Requests to access the datasets should be directed to YZ, 308047491@qq.com.

## Author contributions

XZ and YZ designed the research and methodology and put forward the policy recommendations. XZ provided guidance throughout the entire research process. YL and LL revised and approved the manuscript. All authors contributed to the article and approved the submitted version.
